# ﻿*Renicorisrobustus*, a new genus and species of the subfamily Harpactorinae (Hemiptera, Reduviidae) from China

**DOI:** 10.3897/zookeys.1182.108219

**Published:** 2023-10-20

**Authors:** Jianyun Wang, Zhuo Chen, Ping Zhao, Wanzhi Cai

**Affiliations:** 1 Environment and Plant Protection Institute, Chinese Academy of Tropical Agricultural Sciences, Haikou, Hainan, 571101, China Environment and Plant Protection Institute, Chinese Academy of Tropical Agricultural Sciences Haikou China; 2 Department of Entomology and MOA Key Lab of Pest Monitoring and Green Management, College of Plant Protection, China Agricultural University, Yuanmingyuan West Road, Beijing 100193, China China Agricultural University Beijing China; 3 Key Laboratory of Environment Change and Resources Use in Beibu Gulf (Ministry of Education) and Guangxi Key Laboratory of Earth Surface Processes and Intelligent Simulation, Nanning Normal University, Nanning 530001, China Nanning Normal University Nanning China

**Keywords:** Assassin bug, Euagorasini, key, morphology, new species, taxonomy

## Abstract

*Renicoris***gen. nov.** and its type species *Renicorisrobustus***sp. nov.** (Hemiptera: Heteroptera: Reduviidae: Harpactorinae) from Yunnan, China, are described and illustrated. A key to separate the new genus and its closely related genera is provided.

## ﻿Introduction

Harpactorinae is the largest subfamily of Reduviidae, including more than 2000 species and 300 genera worldwide, and 191 spe`cies and 55 genera in China (Maldonado-Capriles 1990; Zhao et al. 2009, 2014a, b, 2015a, b, 2021; Chen et al. 2020, 2022). However, the tribe-level systematic relationship in Harpactorinae is in debate due to the complex morphological variation and the high biodiversity within the subfamily. The taxonomic status of several harpactorine tribes, Euagorasini Distant, 1904, Rhaphidosomoni Jeannal, 1919, Rhynocorini Villiers, 1982 and Sycanini Dohrn, 1859 are not recognized by all taxonomists (Distant 1904; Hsiao and Ren 1981; Forero 2011). It is worthwhile to study whether some generic groups should be promoted to tribal level.

During fieldwork to Yunnan Province in southwestern China, we discovered an undescribed, rare, and unique species belonging to the tribe Euagorasini. Euagorasini was established by Distant (1904) based on the following characters: body slender; head with a pair of spines or tubercules at the base of the antennae; lateral pronotal angles generally produced into spines. The tribe consists of 29 genera and 76 species in China (Hsiao and Ren 1981; Cai and Tomokuni 2003; Chen et al. 2005; Truong et al. 2005; Zhao et al. 2006a, b, 2014a, 2021; Huang et al. 2007; Chen et al. 2020). The head of the undescribed species has a small round tubercle behind the base of the antennal tubercle, which is an important common character for all genera of Euagorasini (Distant 1904; Hsiao and Ren 1981). We could not assign it to any known genus and have therefore erected a new monotypic genus to accommodate it. A key to the new genus and related genera is provided.

## ﻿Material and method

This study is based on material deposited in the Entomological Museum of China Agricultural University (CAU), Beijing, China. External structures were examined using a binocular dissecting microscope. Male genitalia were soaked in hot 90% lactic acid for ~10 min to remove soft tissue, then rinsed in hot distilled water and dissected under a microscope. Dissected parts of the genitalic structures were placed in a plastic microvial with lactic acid under the corresponding specimen. All habitus photographs were taken using Canon D60 SLR camera (Canon Inc., Tokyo, Japan). All photographs of the male genitalia were taken with the aid of the Research Stereo Microscope SMZ25 (Nikon Corporation, Tokyo, Japan). Measurements were obtained using a calibrated micrometer; body length was measured from the apex of the head to the tip of the fore wings in a resting position; maximum width of the pronotum was measured across humeral angles. All measurements are given in millimeters. Morphological terminology and the classification system mainly followed those of Cai and Tomokuni (2003), Davis (1966) and Hsiao and Ren (1981).

## ﻿Taxonomy

### ﻿Subfamily Harpactorinae Amyot & Servile, 1843

**Tribe Euagorasini Distant, 1904**

#### ﻿ Renicoris gen. nov.

Taxon classificationAnimaliaHemipteraReduviidae

D77D0054-F02D-5048-B3DA-81236882B5A4

https://zoobank.org/96E69160-7238-483C-8CD0-22F155C20036

Figs 1 , 2 , 3

##### Type species.

*Renicorisrobustus* sp. nov.

##### Type locality.

China, Yunnan, Lvchun, Huanglian Mountain.

##### Diagnosis.

**Figure 1.**
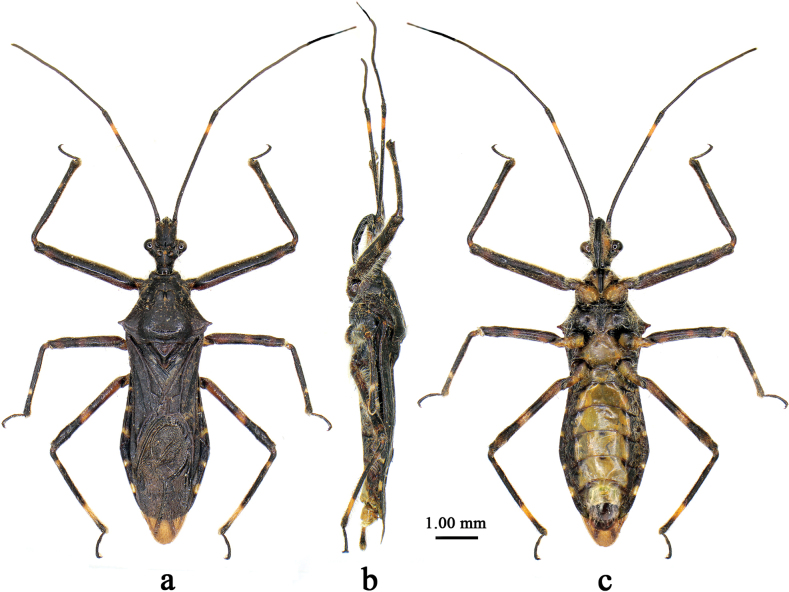
*Renicorisrobustus* sp. nov., male, holotype, habitus **a** dorsal view **b** lateral view **c** ventral view.

**Figure 2.**
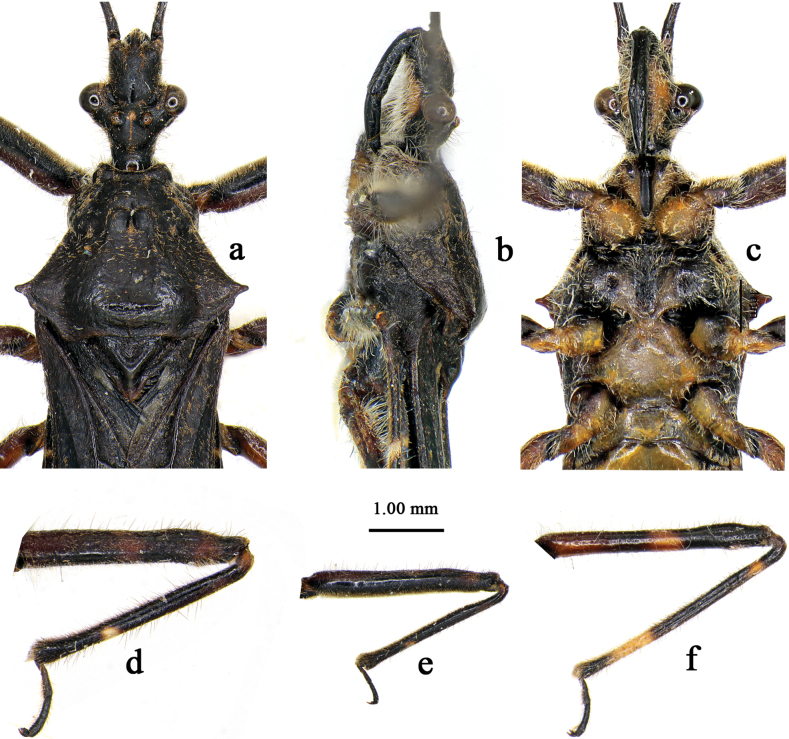
*Renicorisrobustus* sp. nov., male, holotype **a–c** head and pronotum, with antennae and legs not shown **d** fore leg **e** mid leg **f** hind leg. **a** dorsal view **b, d–f** lateral view **c** ventral view.

**Figure 3.**
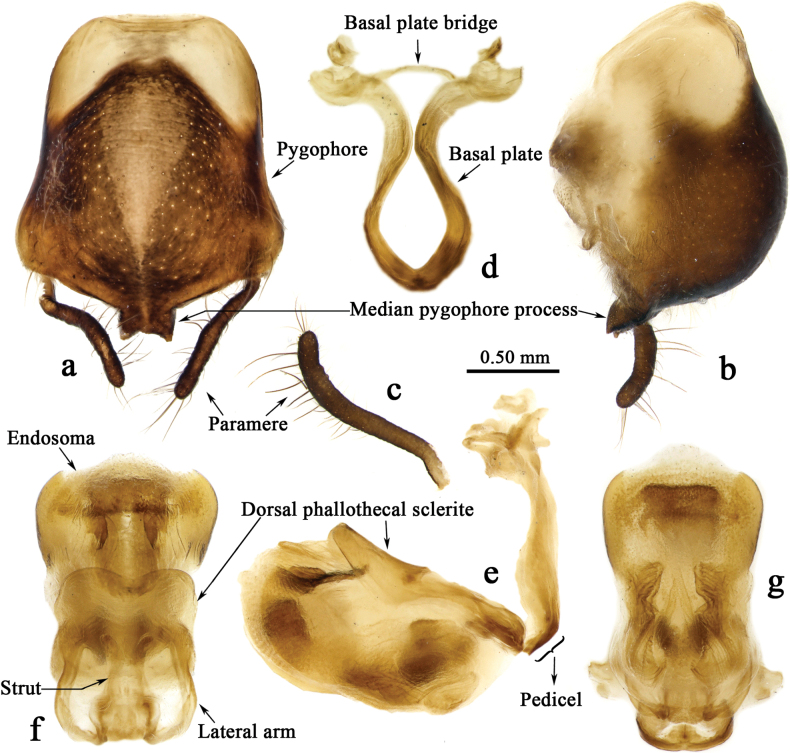
*Renicorisrobustus* sp. nov., male, holotype, genitalia **a** pygophore with two parameres **b** pygophore with a paramere previously extracted **c** paramere **d** phallobase **e** phallus **f, g** phallosoma **a, g** ventral view **e, b** lateral view **f** dorsal view.

*Renicoris* gen. nov. resembles *Chenicoris* Chen & Cai, 2020 in the structure of the head and the male genitalia (Figs 1–3). However, in the new genus, the pronotum is trapezoidal and its median transversal constriction is indistinct (Figs 1, 2); the posterior pronotal lobe is not enlarged, the lateral pronotal angle is spine-shaped; the lateral margin of pronotum is straight (Figs 1, 2); the abdomen is rhomboid, and the fourth to sixth connexival segments of the abdomen are produced laterally (Fig. 1); the median pygophore process is bifid with acute angles (Fig. 3a, b) (vs. in *Chenicoris*, the pronotum is not trapezoidal due to its median transversal strong constriction; the posterior pronotal lobe is much enlarged and the lateral pronotal angle is rounded; the lateral margin of pronotum is distinctly constricted in the middle; the abdomen is not rhomboid, the fourth to sixth connexival segments of the abdomen are produced laterally, especially the lateral angle of the fifth segment which is dilated and round; the median pygophore process is absent). The genera morphologically related to the new genus can be separated using the following key.

##### Generic character.

Body somewhat robust (Figs 1, 2). Head shorter than pronotum, with a small round tubercle behind base of each antennal tubercle (Figs 1a, 2a); eyes large and protruded laterally (Figs 1, 2a–c); ocelli elevated; anteocular part slightly longer than postocular part, transversely constricted between eyes; postocular part posteriorly narrower (Figs 1, 2a–c); first antennal segment nearly as long as head and pronotum together in length; first rostral segment longest and extending to middle of eyes (Fig. 2b). Pronotum dorsally slightly flat, somewhat anteriorly declining, medially with indistinct transversal constriction; lateral margin nearly straight; anterior angle round; anterior pronotal lobe 1/2 as long as posterior lobe; middle part of posterior lobe faintly bulgy, two sides with lateral sulci; lateral pronotal angles produced laterally, short spine-shaped, with round protuberance behind it; posterior and posterolateral margins nearly straight; posterior angle round; scutellum subtriangular with Y-shaped ridge (Figs 1, 2a–c). Legs thick and robust, fore legs somewhat thickened (Figs 1, 2). Fore wing with inner cell wider than outer cell at base. Fourth to sixth connexival segments of abdomen laterally slightly rhombus-shaped dilated.

##### Distribution.

China (Yunnan).

##### Etymology.

The genus is named after the Chinese entomologist Shu-Zhi Ren (Nankai University, Tianjin, China), for her great contribution to the taxonomy of Chinese Heteroptera. The Greek noun *coris* means “bug”. Gender masculine.

### ﻿A key to *Renicoris* gen. nov. and its morphologically similar genera

**Table:** 

1	Apical part of fore tibia distinctly bent	**2**
–	Apical part of fore tibia straight	**5**
2	Fore femur prominently thickened and robust	***Agyrius* Stål, 1863**
–	Fore femur somewhat thickened	**3**
3	Inner side of subapical part of fore tibia armed with a long spur	***Rihirbus* Stål, 1861**
–	Fore tibia unarmed	**4**
4	Pronotum conspicuously anteriorly declining; posterior pronotal lobe anteriorly faintly elevated, and two sides without lateral sulci	***Flexitibia* Zhao & Cai, 2014**
–	Pronotum not declining; posterior pronotal lobe not elevated, middle part feebly concave and two sides with lateral sulci	***Camptibia* Cai & Tomokuni, 2003**
5	Posterior part of lateral margin of anterior pronotal lobe with a distinct protuberance	***Isyndus* Stål 1858**
–	Lateral margin of pronotum without protuberance	**6**
6	Fourth to sixth connexival segments of abdomen laterally dilated, fifth connexival segment prominently roundly-produced laterally	***Chenicoris* Chen & Cai, 2020**
–	Fourth to sixth connexival segments of abdomen laterally slightly rhombus-shaped, dilated	***Renicoris* gen. nov.**

#### ﻿ Renicoris robustus sp. nov.

Taxon classificationAnimaliaHemipteraReduviidae

BCC33CD6-F459-5A40-94EF-B15B94BBF3AB

https://zoobank.org/C1D2BEB1-1835-4FA8-82BE-EEE732D93AB9

Figs 1 , 2 , 3

##### Type material.

***Holotype*** (♂): China, Yunnan, Lvchun, Huanglian Mountain, Yakou, Yijiao Center, 22°53'48.9"N, 102°18'23.4"E, 1938 m, 2015-VI-8, Jianyun Wang leg. (CAU).

##### Diagnosis.

As for the genus by monotypy.

##### Description.

Macropterous male. ***Coloration*.** Body dorsally bluish-black to black with milky white to yellowish markings, ventrally paler (Fig. 1a–c). Ventral surface of head (Figs 1c, 2c), one small round spot of vertex, ocellus (Figs 1a, 2a), one distinct annular marking of subapical part of first antennal segment (Fig. 1a–c), coxae, trochanters (Figs 1c, 2c), three faint annular markings of basal, median and apical parts of fore and mid femora (Figs 1, 2d, e), one distinct annular marking of median part and two faint markings of basal and apical parts of hind femera (Figs 1, 2f), one faint marking of basal part and one distinct marking of subapical part of fore and mid tibiae (Figs 1, 2d, e), one distinct annular marking of subapical part and one small marking of subbasal part of hind tibiae (Fig. 2f), sterna of pro- and metathoraxes (Figs 1c, 2c), sterna of abdomen (except connexivum and lateral margins) (Fig. 1c), and markings of posterior margins of connexival segments, milky white to yellowish (Fig. 1).

***Structure*.** Body of medium size, somewhat flattened dorsoventrally (Fig. 1). Head, thorax, abdomen, and legs covered with white short bent procumbent pubescence and sub-erect setae; first antennal segment sparsely clothed with erect setae, second to fourth segments densely clothed with procumbent pubescence; fore femur and tibia ventrally clothed with dense short setae (Figs 1, 2). Head width subequal to or slightly shorter than length; interocular space more than 2× interocellar space; rostrum robust, first segment subequal to second and third segments together in length (Fig. 2b, c). Anterior pronotal lobe basally centrally sulcate and laterally with shallow arc-shaped glabrous area; middle part of posterior pronotal lobe somewhat concaved; lateral pronotal angles acutely produced, short (Figs 1a, 2a). Fore wing surpassing abdominal tip by 0.7 mm (Fig. 1).

***Male genitalia*.** Pygophore oblong, median pygophore process bifid with acute angles (Fig. 3a, b); paramere clavate, slightly curved, middle part twisted (Fig. 3a–c); basal plate of phallobase longer and thicker than basal plate bridge, pedicel short (Fig. 3d, e). Phallosome elliptical (Fig. 3e–g); dorsal phallothecal sclerite well sclerotized, apical part concave, lateral arm subequal to strut in length (Fig. 3e); apical part of endosome armed with a pair of leaflike sclerites (Fig. 3e, f).

##### Measurements

[**male (*N* = 1), in mm].** Body length 14.4 (to tip of abdomen) / 15.1 (to tips of fore wings). Length of head 2.6 (with neck) / 2.4 (without neck); length of anteocular part 1.0; length of postocular part 0.75; width across eyes 2.1; interocular space 1.2; interocellar space 0.45; length of antennal segments I–IV 5.5, 2.0, 3.2, 1.8; length of rostral segments I–III 1.4, 0.9, 0.5. Length of anterior pronotal lobe 1.2; length of posterior pronotal lobe 2.0; length of pronotum 3.2; width of anterior pronotal lobe 2.2; width of posterior pronotal lobe 4.3; basal width of scutellum 1.6; median length of scutellum 1.1; length of fore wing 9.9; length of fore femur / tibia / tarsus 4.9 / 4.5 / 1.1; length of mid femur / tibia / tarsus 3.5 / 3.2 / 1.1; length of hind femur / tibia / tarsus 4.7 / 4.7 / 1.1. Length of abdomen 7.2; maximum width of abdomen 4.2.

##### Distribution.

China (Yunnan).

##### Etymology.

The specific name alludes to the robust body shape of the new species. The Latin noun *robustus* means “sturdy, strong”.

##### Biology.

Unknown.

## Supplementary Material

XML Treatment for Renicoris

XML Treatment for Renicoris robustus

## ﻿Additional information

### Author ORCIDs

Jianyun Wang https://orcid.org/0000-0002-8466-3252

Zhuo Chen https://orcid.org/0000-0003-3393-0338

Ping Zhao https://orcid.org/0000-0001-9315-2756

### Data availability

All of the data that support the findings of this study are available in the main text.
